# Agro-Morphological Characterization of Sicilian Chili Pepper Accessions for Ornamental Purposes

**DOI:** 10.3390/plants9101400

**Published:** 2020-10-21

**Authors:** Giuseppe Virga, Mario Licata, Beppe Benedetto Consentino, Teresa Tuttolomondo, Leo Sabatino, Claudio Leto, Salvatore La Bella

**Affiliations:** 1 Research Consortium for the Development of Innovative Agro-Environmental Systems (CoRiSSIA), Via della Libertà 203, 90143 Palermo, Italy; giuseppe251@hotmail.it (G.V.); claudio.leto@unipa.it (C.L.); 2 Department of Agricultural, Food and Forest Sciences, Università degli Studi di Palermo, Viale delle Scienze 13, Building 4, 90128 Palermo, Italy; mario.licata@unipa.it (M.L.); beppebenedetto.consentino@unipa.it (B.B.C.); salvatore.labella@unipa.it (S.L.B.)

**Keywords:** chili pepper, ornamental plants, *Capsicum* genera, local genetic variability, characterization

## Abstract

The species belonging to the genus *Capsicum* have been widely used as decorative vegetables, however only a few genotypes are available for this purpose. The goal of the present work was the agro-morphological characterization of several chili pepper accessions cultivated into different pot sizes (10, 14, 18 or 20 cm diameters). The agro-morphological characterization of 19 accessions was performed following IPGR (International Plant Genetic Resources Institute) descriptors: plant height (PH), plant canopy width (PCW), PH/PCW ratio, plant growth habit, plant visual quality, first flower emission, fruiting start, end of harvest, fruit number, fruit length, fruit width, fruit color at mature stage and fruit shape. Analysis of Variance (ANOVA) for all observed traits showed statistical significant differences among the genotypes tested. Results of the heat map complementarily secured the frequency of multiplicity highlighted from the ANOVA analysis. Furthermore, the present study pointed out that A33, A27, G1 and A1 chili pepper accessions achieved optimal performances in terms of plant visual quality, which is a crucial trait for ornamental purposes.

## 1. Introduction

Chili pepper (*Capsicum* spp.) is a Solanaceous crop cultivated and appreciated all over the world. Within the *Capsicum* genera, five species (*C. annum*, comprising the NuMex, Jalapeño and Bell varieties, *C. frutescens*, including Tabasco variety, *C. chinense*, comprising Habanero and Scotch Bonnet varieties, *C. baccatum* involving the Aji varieties and *C. pubescens*, containing the Rocoto and Manzano varieties) enclose the cultivated varieties [1]. Chili pepper is an important vegetable, mostly for its fruit nutritional and functional features such as capsaicinoids, carotenoids, antioxidant vitamins and phenolic constituents; furthermore, hot pepper fruits are used as food coloring and flavoring [2]. Chili pepper consumption is primarily due to its spicy taste and flavor which derives from the capsaicinoids synthesis (capsaicin and dihydrocapsaicin). It is well documented that capsaicinoids retain essential biological activities, such as physiological, pharmacological and antimicrobial functions. Consequently, chili pepper is recommended for the treatment of numerous distressing and inflammatory disorders [3,4]. However, as reported by Iqbal et al. [5], the quantitative and qualitative traits of chili pepper fruit are linked up with genotype, maturity stage and growth cultivation conditions. Among the above mentioned *Capsicum* ssp, *C. annum* is the species most often grown as an ornamental. Ornamental peppers are mainly marketed as potted plants to produce morphologically and color-diverse peppers. Potted ornamental peppers are generally marketed from September to December, with a lower amount at other times of the year. The range of diversity of fruit size and type, plant height, fruit and foliar color have contributed to the success and widespread acceptance of the pepper in the ornamental plot plant arenas. Efficient sexual propagation, reduced cropping cycle, heat and drought tolerance and vivid fruit colors, have contributed to the success of ornamental peppers. Growers aspire to specific features to boost the cost-effectiveness of ornamental pot peppers. Overall, plants should be compact, with dense foliage, edible colorful fruits characterized by unusual shapes, and they should be drought and disease tolerant. Ornamental peppers with a prostrate growth habit are best suited as bedding, garden plants and hanging baskets [6].

The production of new genotypes characterized by good pot harmony and dimension is one of the main purposes of ornamental pepper breeding programs [7]. Although ornamental peppers are considered self-pollinating, they can be insect-pollinated and, according to Bosland [8], cross pollination can range from 2% to 90%. Pepper nurseries provide at the moment a large list of varieties suitable for pot production. However, plant nurseries involved in ornamental pepper production do not always assure sufficient isolation among plants in order to ensure self-pollination. Therefore, inter cultivar hybridization can occur during the plant propagation process. Our preliminary observation has suggested the presence of significant variation among the genotypes grown on the island of Sicily, probably due to a certain amount of cross pollination.

Genetic diversity research via genotype-clustering methods permits the assessment of the degree of similarity or diversity among genotypes [9], and therefore can be a valuable tool for breeding improvement [10]. Sicily, being an island in the middle of the Mediterranean Sea, has been an important center of origin and differentiation of several fruiting and leafy vegetables [11,12,13,14,15,16]. Indeed, Raimondo et al. [14] estimated 2650 taxa over an area of 26,000 km^2^, comprising both specific and intraspecific taxa. Although Sicily is not the center of genetic diversity for the chili pepper, the long-time cultivation of this American-originating species [17] and the diversity of genetic material available on the island has caused a certain range of genetic variability which could be exploited for further development, especially to select genotypes which could be used in the ornamental sector and eventually become more adapted to the island environment.

Genetic diversity is the basis for increasing the effective utilization of germplasm during a breeding program. Consequently, the number of accessions is a crucial element in the search for new characteristics or characteristics combinations [18]. Thus, starting from the aforementioned considerations, the aim of the present study was the morphological and agronomical characterization of several Sicilian chili pepper accessions. The growing response of these accessions to various pot sizes was also tested based on flowering pot utilization.

## 2. Results

### 2.1. Germplasm Evaluation

Evaluation data are presented in Table S1. Regardless of the accessions, plants grown in 20 and 18 cm diameter pots showed the highest values in terms of PH, whereas plants cultivated in 10 cm pots showed the lowest one (Table S1). Irrespective of the pot size, the A13 accession revealed the highest PH values, followed by the A34 chili pepper accession. The lowest plant height was observed in the A6, A8, A16, A20, A21 and A30 accessions. ANOVA analysis for PH showed a significant interaction between pot size and accession; the highest values were collected in plants belonging to the A5 accession cultivated in pots of 20 cm of diameter, whereas, the lowest PH values were observed in A6 accession plants grown in 10 cm diameter pots (Figure S1). Data collected on PCW supported the trend established for PH (Table S1). However, the interaction plot (Figure S2) showed that the higher values in terms of PCW were observed in A13 accession plants cultivated in 20 cm diameter pots. The lowest values were recorded in plants belonging to the accession A6 grown in 10 cm diameter pots.

Unrelatedly, of the accessions tested, the highest PH/PCW ratio was obtained from plants grown in 10 cm diameter pots, whereas the lowest values were recorded in plants cultivated in 18 and 20 cm diameter pots (Table S1). Disregarding the pot diameter, the A18 accession showed the highest PH/PCW values, followed by A7 and A19 accessions. The A1, A4, A5, A23 and A31 accessions did not differ from the A18, A7 and A19 accessions in terms of PH/PCW. The lowest PH/PCW values were observed in the A6, A8, A15, A16, A17, A20, A21, A27, A29 and A32 accessions. As regards PH/PCW ratio, a significant interaction was found between pot and accession; the plot of interaction (Figure S3) showed the highest values in terms of PH/PCW in the A18 hot pepper accession cultivated in 10 cm diameter pots, whereas the lowest ones were in plants belonging to the A21 accession cultivated in 18 cm diameter pots.

Without regard for the accession, the plants cultivated in 14, 18 and 20 cm diameter pots displayed the best plant visual quality. Regardless of the pot diameter, A29 reached the best visual quality, whereas the A4, A5, A6, A10, A13, A20, A28 and A30 accessions showed the lowest ones. ANOVA for plant visual quality revealed a significant effect of the interaction P × A (Table S1). As reported in the plot of interaction (Figure S4), the best visual quality was detected in the A29 accession grown in 14 cm diameter pots, while the A5, A9, A14 and A30 accessions revealed the lowest values.

### 2.2. Morphological and Agronomical Characterization of 19 Accessions

Considering that two-way ANOVA analysis revealed a significant effect of the year on many recorded traits (PH, PCW, PH/PCW, fruit number, plant visual quality, first flower emission and fruiting start) (Supplementary Table S2), all data sets were also subjected to a one-way ANOVA analysis. Thus, the data were analyzed separately per year. Plant morphological traits are presented in Table 1.

In 2016, the A32 accession had the highest plant height followed by the A24, A1 and A21 accessions, whereas the lowest plant height was observed in A2, A6, A14, A16 and G1 accessions.

As regards PCW, the highest values were recorded in the A17 accession, followed by A25, while the A15, G1 and A32 accessions did not significantly differ from A17 and A25 in terms of PCW. The lowest values were observed in the A18 accession (Table 1).

As regard PH/PCW ratio, A18 showed the highest values, followed by A1, which in turn revealed a higher value than A24. The lowest PH/PCW ratio was recorded in G1.

As concerns the plant growth habit, the A1, A2, A6, A8, A15, A17, A18, A21, A22, A25, A29 and A32 accessions showed an erect habitus, whereas the other genotype tested displayed a compact plant growth habit.

In 2016, the highest scores in terms of plant visual quality were recorded in the A33 accession, followed by A27, which in turn revealed a higher plant visual quality score than A1 and G1. The lowest scores were attributed to the A6 accession. In 2017, the highest plant visual quality scores were recognized in the A25 and A29 genotypes, although the A27 accession showed an interesting ornamental value. It is noteworthy that the data set recorded in 2017 sustained the trends established in 2016 (Table 1). However, two-way ANOVA analysis and means separation (Supplementary Tables S2 and S3) showed a significant effect of the interaction accession × year for PH; the highest values were recorded in the A32 × 2016 combination, followed by the A32 × 2017, A18 × 2017 and A21 × 2017 combinations. The lowest values, in terms of PH, were recorded in the A6 × 2016 combination.

ANOVA analysis for PCW displayed a significant effect of the interaction accession × year (Table S2). As reported in Table S3, the highest PCW values were observed in the A17 accession cultivated in 2017, followed by the A15 × 2016, A32 × 2016 and G1 × 2016 combinations, which in turn revealed higher PCW values than the A21 and A25 accessions grown in 2016 and the A17 accession cultivated in 2017. The lowest PCW values were collected in plants belonging to the combination A6 × 2017.

The highest values in terms of PH/PCW were obtained in the A18 × 2017 combination, whereas the lowest ones were recorded in the combination G1 × 2016.

The best visual quality values were recorded in the A25 and A29 accessions cultivated in 2017 (Table S3), followed by the combinations A33 × 2016, A15 × 2017, A22 × 2017, A27 × 2017 and A32 × 2017. However, visual quality in accession A16 grown in 2017 did not significantly differ from the A25 and A29 accessions cultivated in 2017 or from the A33 × 2016, A15 × 2017, A22 × 2017, A27 × 2017 and A32 × 2017 combinations. The lowest plant visual quality values were collected in the combinations A6 × 2017 and A8 × 2017.

Flowering features and fruit morphological traits recorded in 2016 are presented in Table 2.

The A15 and A29 accessions revealed the earliest first flower emission (29.7 and 31.3 DAT, respectively), followed by the A24 accession, whereas A18, A22, A25 and A27 revealed a late first flower emission (43.0, 42.3, 42.3 and 43.3 DAT, respectively).

Data collected on fruiting start and end of harvest supported the trend established for first flower emission (Table 2).

The A25 accession produced the highest fruit number, followed by the A29 and A32 accessions, whereas the lowest fruit number per plant was recorded in the A8 accession.

The A12 accession produced the longest fruits (5.4 cm) followed by the A16 and A33 accessions (4.7 and 4.3 cm, respectively). The shortest fruits were observed in the A32 genotype (0.8 cm).

The A2 accession showed the highest values in terms of fruit width, followed by the A24 and A8 accessions. The lowest fruit width values were recorded in fruits from the A32 accession (Table 2).

The A21 and A29 accessions produced purple fruits, whereas A27 revealed a dark red fruit color. The A6, A14, A16, A18 and A25 accessions displayed red-colored fruits, while A2, A8, A17, A22, A24 and A32 produced fruits characterized by a light red fruits color. G1 produced orange fruits, whereas the A12, A15 and A33 genotypes showed fruits distinguished by an orange-yellow color. Finally, the A1 accession produced pale orange-yellow fruits (Table 2).

Out of 19 accessions, 6 (A14, A15, A18, A22, A27 and A33) showed a tomato-pepper fruit shape, while A1 and A24 displayed a blocky fruit shape. The A2, A6, A8, G1, A21, A29 and A32 accessions revealed a triangular fruit shape, whereas A17 produced fruits characterized by an almost round shape. Lastly, the A12, A16 and A25 genotypes produced elongated fruit (Table 2). Remarkably, the data set on flowering features and fruit morphological traits recorded in 2017 supported the trend recognized in 2016 (Table 3).

Two-way ANOVA analysis and means separation for the interaction accession × year for first flower emission showed a significant effect (Supplementary Tables S2 and S3); the earliest flower emission was recorded in the A29 accession cultivated in 2017, followed by the A12 accession grown in 2017, whereas the late first flower emission was recorded in the combinations A18 × 2016 and A27 × 2016.

A significant interaction was found between accession × year in terms of fruiting start (Table S2); the earliest fruiting start values were recorded in the combinations A15 × 2017 and A29 × 2017 (Table S3), whereas late fruiting start values were recorded in the A25 accession grown in 2016.

ANOVA analysis showed, also, a significant interaction of accession × year for fruit number (Table S2); the highest numbers of fruits were collected from the A25 × 2016 and A29 × 2016 combinations (Table S3). The combination A8 × 2017 produced the lowest number of fruits.

**Figure 1:**
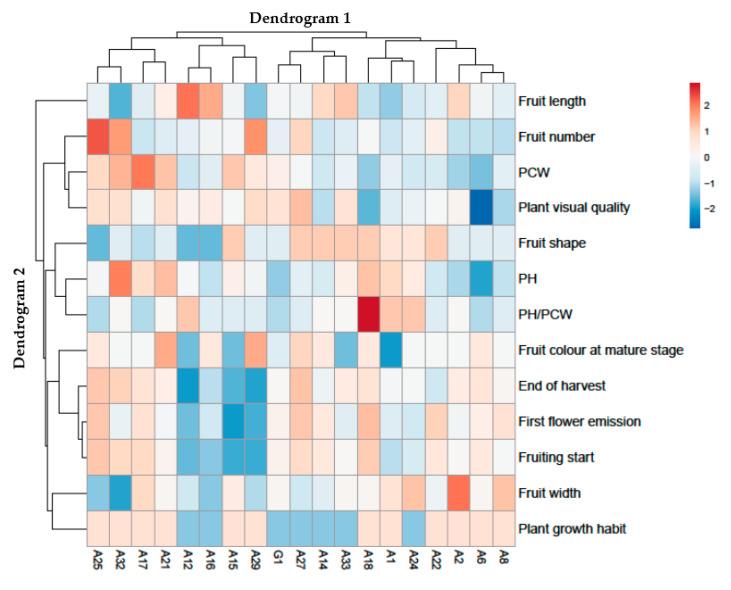
Cluster heat map analysis summarizing two-year (2016 and 2017) Sicilian chili pepper accessions’ responses to agro-morphological characterization by using IPGR (International Plant Genetic Resources Institute) descriptors. The figure was created utilizing the https://biit.cs.ut.ee/clustvis/ online program package with Euclidean distance as the similarity measure and hierarchical clustering with complete linkage. PH: plant height; PCW: plant canopy width.

Climatic data recorded in 2016 and 2017 (Figure 1) revealed that in 2016, from January to July, minimum temperatures were lower than in 2017, whereas from July to November, an inverse trend was recorded. As regards the maximum temperature, in 2016, from January to half of March, climatic data showed lower temperatures than in 2017, while from the middle of March to end of December, the maximum temperatures in 2016 were higher than in 2017.

### 2.3. Heat Map Analysis of All Morphological and Agronomical Descriptors

A grouped data heat-map analysis of the morphological and agronomical descriptors (IPGRI descriptors for Solanaceous) was carried out to show a chromatic appraisal of the different accessions. The heat map analysis showed a couple of dendrograms, the first structured on the top (Dendrogram 1), an arrangement that corresponded to the chili pepper accessions, and the second on the left (Dendrogram 2) showing the IPGR descriptors that affected this distribution. Dendrogram 1 displayed two main groups: on the left, the cluster corresponds to the A25, A32, A17, A21, A12, A16, A15 and A29 accessions, while on the right side of the heat map, the cluster includes the G1, A27, A14, A33, A18, A1, A24, Morando, A2, A6 and A8 chili pepper genotypes (Figure 1).

Particularly, on the left side of Dendrogram 1, two clusters were identified. The first on the left includes the A25, A32, A17 and A21 accessions, separated from A12, A16, A15 and A29, which reveals in particular lower values for fruit shape, PH, fruit color at mature stage, end of harvest, first flower emission, fruiting start, fruit width and plant growth habit, but higher values for fruit length and PH/PCW. The grouping on the left includes the A25 and A32 genotypes. Within this cluster, the A32 accession is evidently divided by higher PH and lower fruit length, fruit number and fruit width, whereas the grouping on the right comprised A17 and A21 chili pepper accessions. Within this cluster, the A17 accession was separated by higher PCW and lower first flower emission, fruiting start and fruit width values.

On the right side of the Dendrogram 1, two clusters were recognized, the first on the left incorporating the G1, A27, A14 and A33 genotypes separately from A18, A1, A24, A22, A2, A6 and A8, which showed, specifically, higher fruit length and fruit shape, but lower plant growth habit values. The grouping on the left includes the G1 and A27 accessions. Within this cluster, the G1 genotype is manifestly separated by lower fruit number, PH, end of harvest, first flower emission, fruiting start and fruit width values, while the grouping on the right embraces the A14 and A33 accessions. Inside this cluster, the A14 accession is divided by lower plant visual quality, PH, and higher fruit color at mature stage. As regard the clusters on the right side of the Dendrogram 1, the grouping on the left includes the A18, A1 and A24 accessions. Within this cluster, the first on the left comprises the A18 genotype, separated from the accessions named A1 and A24, which showed in particular lower PCW and plant visual quality, but higher PH/PCD, first flower emission and fruit starting values. The grouping on the right includes the A22, A2, A6 and A8 accessions. Within this cluster, A22 is clearly separated from the A2, A6 and A8 genotypes by higher fruit number and fruit shape, whereas, the A2 accession is divided from the A6 and A8 genotypes by higher fruit length and fruit width values. Finally, A6 is parted from A8 by lower plant visual quality and PH values. Fascinatingly, the clusters in Dendrogram 2 clearly highlight the differential influences of the different chili pepper accessions.

## 3. Discussion

The contracted multiplicity of ornamental chili pepper forms accessible for commercial purposes in Sicily denotes a challenge to production. The present study was assembled in order to assist the task faced by commercial farmers, and thereby characterize the diversity of local chili pepper genotypes [19]. Hot pepper accessions were characterized by qualitative and quantitative morphological descriptors [20,21], which can also be useful in future breeding programs.

For several traits, a certain degree of variability was observed, and this could be used to categorize the accessions. These features comprised PH, PCW, PH/PCW, plant growth habit, plant visual quality, first flower emission, fruiting start, end of harvest, fruit number, fruit length, fruit width, fruit color at mature stage and fruit shape. Discrepancies in growth and development, quality and yield characteristics were described among pepper genotypes [22,23,24]. Fruit mass and number of fruits per plant are imperative features that directly contribute to yield [25]. Our data revealed different yield traits among the accessions tested. This finding is in accordance with that obtained by Hosamani [26], Rodríguez et al. [27], Manyasa et al. [28] and Sharma et al. [22], who, by conducting a morphological and agronomical characterization of vegetables belonging to the *Capsicum* genera, found high coefficients of variation for fruit mass (22.2%) and number of fruits per plant (36.3%). This suggested a greater scale of unpredictability among genotypes for the aforesaid traits. Our findings are, also, in accordance with those stated by Orobiyi et al. [23], who, studying the agro-morphological characterization of chili pepper landraces grown in northern Benin, found a variable productivity among different classes of chili peppers tested. In this regard, the authors claimed that these results could be explained by the fact that some landraces are very susceptible to wilting and present fruit rot or premature fall. As concerns the yield traits, comparable findings were reported by Lahbib et al. [29] who, when studying the genetic variability of a collection of *Capsicum annuum* landraces, found that yield and yield-related traits were significantly affected by the genotype factor. Furthermore, our results are in line with those reported by Yatung et al. [30], who investigated the morpho-chemical characteristics of chilli pepper genotypes from the Indian continent. According to Bonny [31], the association records are noteworthy in a varied selection given their breeding improvements, as they allow the enhancement of some variables when used minimally. In this respect, Orobiyi et al. [24] reported that yield traits are correlated with vegetative features, such as plant height and leaf width. Remarkably, our results revealed that plant visual quality scores increase with the decrease in the number of fruits per plant. Thus, we might speculate that the variability observed among the dependent variables studied could indicate the intensity of selection for yield or ornamental purposes. In support of these outcomes, the heat map findings reinforced the incidence of diversity evidenced in the ANOVA analysis. Finally, our results also showed that year significantly affected many recorded variables. These outcomes are in line with those reported by Tripodi et al. [32], who confirmed the environmental effects on the agronomic, health-related compounds and antioxidant properties of hot peppers for diverse market destinations.

## 4. Materials and Methods

### 4.1. Germplasm Establishment

The germplasm was established in 2006 at the experimental field of the Department Agriculture, Food and Forestry Sciences of Palermo (SAAF), University of Palermo (longitude 13°21’ E, latitude 38°06’ N, altitude 14 m). Seeds of 34 accessions of ornamental chili peppers collected in various Sicilian farms (A1, A2, A3, A4, A5, A6, A7, A8, A9, A10, A11, A12, A13, A14, A15, A16, A17, A18, A19, A20, A21, A22, A23, A24, A25, A26, A27, A28, A29, A30, A31, A32, A33 and A34) were seeded into 104-cell plug trays containing peat moss (FAP, Padova, Italy). The trays were situated in a greenhouse with a target air temperature of 25/18 °C (day/night). All trays were adequately irrigated. After 55 days, all accessions were transplanted into 10, 14, 18 or 20 cm diameter plastic pots filled with 75:25 (v:v) substrate mix of peat moss (FAP, Padova, Italy) and perlite (Perlite Italiana s.r.l., Milan, Italy). All pots were moved into an open field and placed on a mulched soil with a black polypropylene film. The plants were fertigated via a drip irrigation system and the composition of the nutrient solution was as follow: 0.75 mM NH^4+^,6.5 mM K^+^, 5.0 mM Ca^2+^, 1.5 mM Mg^2+^, 15.5 mM NO_3_^−^, 1.75 mMSO_2_^4−^, 1.25 mMH_2_PO_4_, 15.0 μM Fe, 10.0 μM Mn, 5.0 μM Zn, 30 μM B and 0.75 μM Cu [33].

The experimental field was equipped with drip irrigation systems. During the growing season of plants, the moisture levels in pot substrate were evaluated by measuring the substrate water content percentage, in accordance with Yadav et al. [34]. The water percentage in the substrate was determined at 48 h after the pots were watered to field capacity of the substrate. Three substrate samples of 50 g each were randomly collected from pots for each irrigation water treatment, oven dried at 100 °C until constant weight and, then, reweighed. The substrate water content percentage was calculated using the formula [34]:

$$\mathrm{substrate}\;\mathrm{water}\;\mathrm{content}=\frac{\left(\mathrm{watered}\;\mathrm{substrate}\;\mathrm{weight}-\mathrm{dried}\;\mathrm{substrate}\;\mathrm{weight}\right)}{\mathrm{watered}\;\mathrm{substrate}\;\mathrm{weight}}\times 10$$

Nutrients and water leaching from pots were collected in dishes placed under each pot and the leachate was returned to the substrate before the irrigation water was applied.

Before flowering, all pots were covered with a non-woven film in order to ensure self-pollination. After fruit set phase, the 34 accessions were characterized by using significant morphological descriptors, such as plant height (PH), plant canopy width (PCW), PH/PCW ratio and plant visual quality. Plant visual quality was scored on a 9 to 1 continuous scale, where 9 refers to optimal appearance, 7 to good, 5 to fair (limit of marketability), 3 to fair (useable but not saleable) and 1 to unusable.

### 4.2. Germplasm Characterization

The trial was conducted in 2016 and repeated in 2017 at the experimental field of SAAF, University of Palermo, located at Sciacca, Agrigento Province (longitude 13°07’ E, latitude 37°30’ N, altitude 31 m). Out of the 34 chili pepper accessions, 18 (A1, A2, A6, A8, A12, A14, A15, A16, A17, A18, A21, A22, A24, A25, A27, A29, A32 and A33) from the previous screening (without synonyms), plus a new one (G1), were seeded and grown as described in the previous trial conducted in 2006. All accessions were self-pollinated from 2006 to the beginning of the germplasm characterization. After 55 days, chili pepper seedlings were transplanted in round plastic pots (18 cm diameter) filled with a 75:25 (v:v) substrate mix of peat moss (FAP, Padova, Italy) and perlite (Perlite Italiana s.r.l., Milan, Italy). The cultivation conditions adopted were the same as those described above. For the morphological and agronomical characterization, the IPGRI descriptors for Solanaceous were adopted [20,35]. Thus, 13 characteristics were evaluated: plant height (PH) (cm), plant canopy width (PCW) (cm), PH/PCW ratio, plant growth habit (5 compact; 7 erect), plant visual quality (from 9 to 1 continuous scale, where 9 refers to optimal appearance, 7 to good, 5 to fair (limit of marketability), 3 to fair (useable but not saleable) and 1 to unusable), first flower emission (days after transplanting (DAT)), fruiting start (DAT), end of harvest (DAT), fruit number (no.·plant^−1^), fruit length (cm), fruit width (cm), fruit color at mature stage (1 white, 3 pale orange-yellow, 4 orange-yellow, 5 pale orange, 6 orange, 7 light red, 8 red, 9 dark red and 10 purple) and fruit shape (1 elongate, 2 almost round, 3 triangular, 4 campanulate, 5 blocky, 6 tomato-pepper, 7 ellipse, and 8 scotch bonnet).

**Figure 2:**
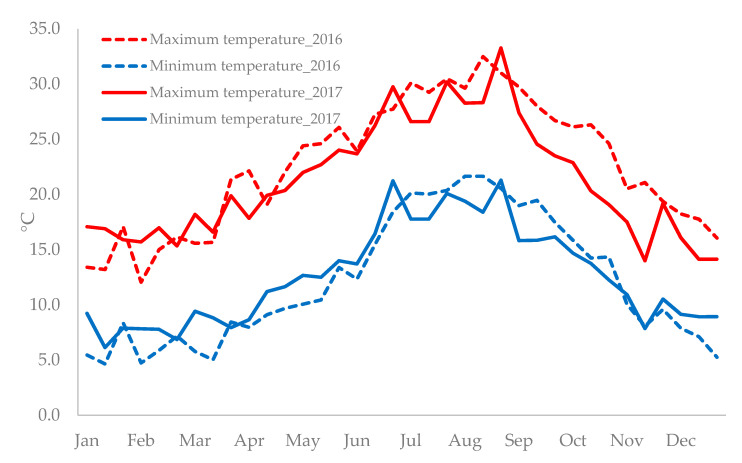
Minimum and maximum air temperature at Sciacca, Agrigento province (longitude 13°07′ E, latitude 37°30′ N, altitude 31 m) during the growing cycles (2016 and 2017).

Climatic data were collected by the meteorological station located at the experimental site. The maximal and minimal temperature in the course of the plant growth cycles were recorded (Figure 2).

### 4.3. Experimental Design and Statistical Analysis

The 34 chili pepper accessions (A1, A2, A3, A4, A5, A6, A7, A8, A9, A10, A11, A12, A13, A14, A15, A16, A17, A18, A19, A20, A21, A22, A23, A24, A25, A26, A27, A28, A29, A30, A31, A32, A33 and A34) were grown in different pot sizes (10, 14, 18 or 20 cm of diameter) in a two-factor experimental design rendering 136 treatments. Each treatment was replicated three times and enclosed 10 plants each, accounting for a total of 4080 hot pepper plants. The effect of the different treatments was evaluated by Analysis of Variance (ANOVA) and the mean separation was accomplished by Tukey HSD test (*p* < 0.05).

For the morphological and agronomical characterization of the 19 accessions (A1, A2, A6, A8, A12, A14, A15, A16, A17, A18, G1, A21, A22, A24, A25, A27, A29, A32 and A33), the source of variance (accessions) was organized in a randomized complete block design with three replicates consisting of 10 pot plants per accession. A preliminary data analysis was conducted by using a two-way ANOVA analysis (accession × year), in order to evaluate the statistically significant effect of the year. However, since ANOVA analysis revealed a significant effect of the year for many of the examined variables, in order to better understand the trend established among the accessions, all the data sets were analyzed by one-way ANOVA. The significance level *p* < 0.05 was employed, and the significant differences between means were appraised using Tukey’s HSD test.

A heat map summarizing all the morphological and agronomical descriptors of chili pepper to different accessions was, also, created using the online program package (https://biit.cs.ut.ee/clustvis/) with Euclidean distance as the similarity measure and hierarchical clustering with complete linkage.

## 5. Conclusions

The present study pointed out that although Sicily is not the center of genetic origin for chili peppers, Sicilian accessions denoted a consistent range of genetic variability. Our study also highlighted that the A33, A27, G1 and A1 chili pepper accessions showed good performance in terms of plant visual quality score. This is an essential prerequisite for ornamental purposes, and therefore these accessions deserve specific attention for future screening activities, breeding improvements and eventual cultivation.

## Figures and Tables

**Table 1:** Effect of 19 chili pepper accessions on plant height (PH), plant canopy width (PCW), PH/PCW ratio, plant growth habit and plant visual quality, in two years (2016 and 2017).

	2016										2017									
Source of Variance	PH (cm)		PCW (cm)		PH/PCW		Plant Growth Habit (5–7)		Plant Visual Quality (1–9)		PH (cm)		PCW (cm)		PH/PCW		Plant Growth Habit (5–7)		Plant Visual Quality (1–9)	
A1	34.0	bc	34.5	efgh	0.99	b	7.0	a	7.2	abc	33.8	bc	34.6	cd	0.98	bcd	7	a	5.5	fgh
A2	20.8	g	28.8	hi	0.72	def	7.0	a	6.9	abcd	22.2	ef	26.8	defg	0.83	cdef	7	a	6.6	cdef
A6	20.2	g	29.5	ghi	0.69	defg	7.0	a	4.0	g	13.0	g	21.8	h	0.59	g	7	a	5.0	gh
A8	21.9	fg	35.5	efg	0.62	efg	7.0	a	6.4	cdef	22.7	ef	32.6	de	0.70	fg	7	a	5.1	gh
A12	24.8	efg	32.3	fghi	0.77	de	5.0	b	6.5	cdef	31.4	cd	27.8	def	1.13	b	5	b	7.2	bcde
A14	21.6	g	32.9	fghi	0.65	defg	5.0	b	5.8	def	27.3	de	29.5	defg	0.93	bcde	5	b	6.0	efg
A15	28.4	cde	54.0	ab	0.53	gh	7.0	a	5.8	def	31.8	cd	38.8	bc	0.82	cdefg	7	a	7.6	abcd
A16	21.3	g	36.8	def	0.58	fg	5.0	b	5.8	def	23.8	ef	30.8	def	0.77	cdefg	5	b	8.2	ab
A17	31.8	bcd	56.7	a	0.56	fgh	7.0	a	7.1	abcd	34.9	abc	49.2	a	0.71	efg	7	a	6.2	efg
A18	32.1	bcd	27.5	i	1.17	a	7.0	a	6.3	cdef	39.3	a	26.3	fgh	1.49	a	7	a	4.6	h
G1	21.2	g	54.0	ab	0.39	h	5.0	b	7.2	abc	19.2	f	24.6	gh	0.78	cdefg	5	b	7.2	bcde
A21	33.7	bc	48.7	bc	0.69	defg	7.0	a	7.1	abcd	38.3	ab	45.3	a	0.85	cdef	7	a	7.5	abcd
A22	23.9	efg	33.2	fghi	0.72	def	7.0	a	5.8	f	23.8	ef	31.3	def	0.76	defg	7	a	7.6	abcd
A24	34.7	b	35.9	ef	0.97	bc	5.0	b	6.4	cdef	26.8	de	26.8	defg	1.00	bc	5	b	6.6	cdef
A25	29.3	bcde	50.2	b	0.58	fg	7.0	a	6.2	def	26.5	de	38.7	bc	0.68	fg	7	a	8.4	a
A27	25.0	efg	40.7	de	0.62	efg	5.0	b	7.7	ab	26.6	de	33.7	cd	0.79	cdefg	5	b	7.8	abc
A29	27.7	def	42.8	cd	0.65	defg	7.0	a	6.3	cdef	27.5	de	39.1	bc	0.71	efg	7	a	8.4	a
A32	41.6	a	52.5	ab	0.79	cde	7.0	a	6.8	bcde	38.9	ab	43.4	ab	0.90	cdef	7	a	7.8	abc
A33	29.2	bcde	35.9	ef	0.81	bcd	5.0	b	7.8	a	30.3	cd	34.8	cd	0.88	cdef	5	b	6.5	def
*Significance*	***		***		***		***		***		***		***		***		***		***	

Data within a column followed by the same letter are not significantly different at *p* ≤ 0.05 according to Tukey HSD Test. The significance is designated by asterisks as follows: ***, statistically significant differences at *p*-value below 0.001.

**Table 2:** Effect of 19 chili pepper accessions on first flower emission, fruiting start, end of harvest fruit number, fruit length, fruit width, fruit color at mature stage and fruit shape, in 2016.

Source of Variance	First Flower Emission (DAT)		Fruiting Start (DAT)		End of Harvest (DAT)		Fruit Number (# Plant^−1^)		Fruit Length (cm)		Fruit Width (cm)		Fruit Color at Mature Stage (1–10)		Fruit Shape (1–8)	
A1	35.7	fg	42.0	gh	79.0	de	132.5	i	1.3	hij	1.27	abcde	3.0	g	5.0	b
A2	37.7	de	48.3	ef	82.0	bcd	109.8	ij	4.1	abcd	1.77	a	7.0	d	3.0	c
A6	39.7	bc	50.3	cde	84.0	b	104.0	ij	2.7	efg	1.13	abcdef	8.0	c	3.0	c
A8	40.7	b	48.7	def	80.0	cde	78.4	j	2.4	fghi	1.50	abc	7.0	d	3.0	c
A12	31.7	h	39.0	ij	63.7	g	265.6	g	5.4	A	0.83	bcdef	4.0	f	1.0	e
A14	40.0	bc	51.0	bcd	78.3	e	137.9	i	4.0	bcde	0.93	bcdef	8.0	c	6.0	a
A15	29.7	i	37.7	j	65.3	g	345.6	f	2.7	defg	1.20	abcde	4.0	f	6.0	a
A16	35.0	g	40.7	hi	71.3	f	325.4	f	4.7	ab	0.63	ef	8.0	c	1.0	e
A17	40.7	b	53.3	ab	84.3	b	113.1	i	2.3	fghi	1.37	abcd	7.0	d	2.0	d
A18	43.0	a	53.7	a	84.7	b	350.3	f	1.7	ghij	1.10	abcdef	8.0	c	6.0	a
G1	39.0	cd	48.7	def	79.3	de	273.2	g	2.8	defg	1.07	abcdef	6.0	e	3.0	c
A21	37.7	de	48.3	ef	82.0	bcd	225.4	h	3.3	cdef	1.07	abcdef	10.0	a	3.0	c
A22	42.3	a	50.7	cde	73.3	f	428.1	e	2.4	fghi	1.03	bcdef	7.0	d	6.0	a
A24	35.0	g	43.7	g	79.7	de	245.9	gh	2.0	fghij	1.53	ab	7.0	d	5.0	b
A25	42.3	a	54.3	a	89.3	a	963.6	a	2.5	fgh	0.60	ef	8.0	c	1.0	e
A27	43.3	a	53.3	ab	89.3	a	621.6	d	2.7	defg	0.80	cdef	9.0	b	6.0	a
A29	31.3	h	37.7	j	63.7	g	834.0	b	1.1	ij	0.67	def	10.0	a	3.0	c
A32	36.7	ef	52.7	abc	88.0	a	793.7	c	0.8	J	0.43	f	7.0	d	3.0	c
A33	36.0	fg	47.3	f	83.0	bc	224.0	h	4.3	abc	1.10	abcdef	4.0	f	6.0	a
*Significance*	***		***		***		***		***		***		***		***	

Data within a column followed by the same letter are not significantly different at *p* ≤ 0.05 according to Tukey HSD Test. The significance is designated by asterisks as follows: ***, statistically significant differences at *p*-value below 0.001.

**Table 3:** Effect of 19 chili pepper accessions on first flower emission, fruiting start, end of harvest fruit number, fruit length, fruit width, fruit color at mature stage and fruit shape, in 2017.

Source of Variance	First Flower Emission (DAT)		Fruiting Start (DAT)		End of Harvest (DAT)		Fruit Number (# Plant^−1^)		Fruit Length (cm)		Fruit Width (cm)		Fruit Color at Mature Stage (1–10)		Fruit Shape (1–8)	
A1	33.0	g	38.7	i	76.7	fg	97.7	h	1.3	hij	1.4	abcde	3.0	g	5.0	b
A2	34.3	f	44.3	fg	79.7	cde	81.6	hi	4.1	abcd	1.8	a	7.0	d	3.0	c
A6	36.0	e	47.0	cde	81.7	bc	76.7	hi	2.7	defg	1.1	abcdef	8.0	c	3.0	c
A8	38.0	c	43.3	gh	77.7	defg	64.9	i	2.4	fghi	1.5	ab	7.0	d	3.0	c
A12	29.0	i	36.0	j	59.7	j	197.5	f	5.4	a	0.8	bcdef	4.0	f	1.0	e
A14	37.0	d	46.3	def	75.0	g	102.4	h	4.0	bcde	0.9	bcdef	8.0	c	6.0	a
A15	27.0	j	34.7	j	63.7	i	252.6	e	2.7	defg	1.2	abcde	4.0	f	6.0	a
A16	32.0	h	36.7	ij	68.7	h	241.7	e	4.7	ab	0.6	ef	8.0	c	1.0	e
A17	38.0	c	49.3	ab	81.7	bc	84.5	hi	2.4	fghi	1.4	abcd	7.0	d	2.0	d
A18	40.0	a	50.7	a	82.0	bc	262.8	e	1.7	ghij	1.1	abcdef	8.0	c	6.0	a
G1	35.7	e	46.0	def	73.3	defg	204.6	f	2.8	defg	1.1	abcdef	6.0	e	3.0	c
A21	34.7	f	45.3	defg	79.0	cdef	263.4	g	3.3	cdef	1.1	abcdef	10.0	a	3.0	c
A22	39.0	b	47.3	bcd	69.7	h	317.4	d	2.4	fghi	1.0	bcdef	7.0	d	6.0	a
A24	32.0	h	41.3	h	76.3	efg	187.8	fg	2.0	fghij	1.5	ab	7.0	d	5.0	b
A25	40.0	a	50.7	a	85.0	ab	722.7	a	2.5	fgh	0.6	ef	8.0	c	1.0	e
A27	39.0	b	48.7	abc	86.3	a	474.5	c	2.7	defg	0.8	bcdef	9.0	b	6.0	a
A29	27.7	j	34.7	j	61.7	ij	618.8	b	1.1	ij	0.7	def	10.0	a	3.0	c
A32	34.0	f	50.0	a	84.3	ab	601.9	b	0.8	j	0.4	f	7.0	d	3.0	c
A33	33.0	g	45.0	efg	80.0	cd	161.4	g	4.3	abc	1.1	abcdef	4.0	f	6.0	a
*Significance*	***		***		***		***		***		***		***		***	

Data within a column followed by the same letter are not significantly different at *p* ≤ 0.05 according to Tukey HSD Test. The significance is designated by asterisks as follows: ***, statistically significant differences at *p*-value below 0.001.

## Supplementary Materials

The following are available online at https://www.mdpi.com/2223-7747/9/10/1400/s1, Table S1: Analysis of variance and mean comparisons for plant height (PH), plant canopy width (PCW), PH/PCW ratio and plant visual quality of 34 chili pepper accessions grown into different pot dimensions. Table S2: Significance of two-way ANOVA analysis.

## References

[1] Conforti F., Statti G.A., Menichini F. (2007). Chemical and biological variability of hot pepper fruits (*Capsicum annuum* var. *acuminatum* L.) in relation to maturity stage. Food Chem..

[2] Bianchi G., Lo Scalzo R. (2018). Characterization of hot pepper spice phytochemicals, taste compounds content and volatile profiles in relation to the drying temperature. J. Food Biochem..

[3] Dorantes L., Colmenero R., Hernandez H., Mota L., Jaramillo M.E., Fernandez E., Solano C. (2000). Inhibition of growth of some food borne pathogenic bacteria by *Capsicum annuum* extracts. Int. J. Food Microbiol..

[4] Perucka I., Materska M. (2001). Phenylalanine ammonia-lyase and antioxidant activities of lipophilic fraction of fresh pepper fruits *Capsicum annuum* L.. Innov. Food Sci. Emer..

[5] Iqbal Q., Amjad M., Asi M.R., Ariño A. (2013). Characterization of capsaicinoids and antioxidants in hot peppers as influenced by hybrid and harvesting stage. Plant. Food Hum. Nutr..

[6] Stommel J.R., Bosland P.W. (2007). Ornamental pepper. Flower Breeding and Genetics.

[7] Nascimento M.F., Nascimento N.F.F., Rêgo E.R., Bruckner C.H., Finger F.L., Rêgo M.M. Genetic diversity in a structured family of six generations of ornamental chili peppers (*Capsicum annuum*). Proceedings of the XXV International EUCARPIA Symposium Section Ornamentals: Crossing Borders.

[8] Bosland P.W. (1993). An effective plant field cage to increase the production of genetically pure chile (Capsicum spp.) seed. HortScience.

[9] Alvares R.C. (2011). Divergência Genética Entre Acessos de Capsicum Chinense Jacq. Coletados no Sudoeste Goiano. Master’s Thesis.

[10] Rêgo E.R., Rêgo M.M., Cruz C.D., Finger F.L., Amaral D.S.S.L. (2003). Genetic diversity analysis of peppers: A comparison of discarding variables methods. Crop. Breed. Appl. Biotechnol..

[11] D’Anna F., Sabatino L. (2013). Morphological and agronomical characterization of eggplant genetic resources from the Sicily area. J. Food Agric. Environ..

[12] Sabatino L., Palazzolo E., D’Anna F. (2013). Grafting suitability of Sicilian eggplant ecotypes onto Solanum torvum: Fruit composition, production and phenology. J. Food Agric. Environ..

[13] Sabatino L., Iapichino G., Maggio A., D’anna E., Bruno M., D’Anna F. (2016). Grafting affects yield and phenolic profile of Solanum melongena L. landraces. J. Integr. Agric..

[14] Raimondo F.M., Gianguzzi L., Ilardi V. (1992). Inventario delle specie “a rischio” nella flora vascolare della Sicilia. Quad. di Bot. Ambient. Appl..

[15] Tuttolomondo T., Dugo G., Leto C., Cicero N., Tropea A., Virga G., Leone R., Licata M., La Bella S. (2015). Agronomical and chemical characterisation of *Thymbra capitata* (L.) Cav. biotypes from Sicily, Italy. Nat. Prod. Res..

[16] Tuttolomondo T., Dugo G., Ruberto G., Leto C., Napoli E.M., Potortì A.G., Fede M.R., Virga G., Leone R., D’Anna E. (2015). Agronomical evaluation of Sicilian biotypes of *Lavandula stoechas* L. spp. stoechas and analysis of the essential oils. J. Essent. Oil Res..

[17] Paik S.Y., Soo R.K., Chang I.S., Chang P.Y., Sung P.H., Suk B.H., Yun W.J., Choi W.J. (2003). Purification and Characterization of Complement-activating Acidic polysaccharides from the Fruits of *Capsicum annuum*. J. Biochem. Mol. Biol..

[18] Ramanatha R.V., Hodgkin T. (2002). Genetic diversity and conservation and utilization of plant genetic resources. Plant. Cell Tissue Organ..

[19] Bosland P.W., Votava E.J. (2012). Peppers: Vegetable and Spice Capsicums.

[20] IPGRI, AVRDC, CATIE (1995). Descriptors for Capsicum (Capsicum spp.).

[21] Engle L.M. Characterization of germplasm. Proceedings of the Vegetable Germplasm Conservation and Management, Organized by the Asian Vegetable Research and Development Center-African Regional Program.

[22] Sharma V.K., Semwal C.S., Uniyal S.P. (2010). Genetic variability and character association analysis in bell pepper (*Capsicum annuum* L.). J. Hortic. For..

[23] Orobiyi A., Loko L.Y., Sanoussi F., Agré A.P., Korie N., Gbaguidi A., Dansi A. (2018). Agro-morphological characterization of chili pepper landraces (*Capsicum annuum* L.) cultivated in Northern Benin. Genet. Resour. Crop. Ev..

[24] Orobiyi A., Loko Y.L., Adjatin A., Sanoussi F., Gbaguidi A., Dansi A., Sanni A. (2015). Horticultural practices and varietal diversity of chili pepper (*Capsicum annuum* L.) in Central and Northern Benin. Genet. Resour. Crop. Evol..

[25] Bozokalfa M.K., Kilic M. (2010). Mathematical modeling in the estimation of pepper (*Capsicum annuum* L.) fruit volume. Chil. J. Agric. Res..

[26] Hosamani R.M. (2003). Variability, correlation and path analysis in kharif grown chilli (*Capsicum annuum* L.) genotypes for different characters. Capsicum Eggplant Newsl..

[27] Rodríguez Y., Depestre T., Gómez O. (2008). Efficiency of selection in pepper lines (*Capsicum annuum*), from four sub-populations, in characters of productive interest. Cienc. Investig. Agrar..

[28] Manyasa E.O., Silim S.N., Christiansen J.L. (2009). Variability patterns in Ugandan pigeonpea landraces. J. SAT Agric. Res..

[29] Lahbib K., Bnejdi F., El Gazzah M. (2013). Selection of pepper parent from a collection of *Capsicum annuum* landraces based on genetic diversity. J. Plant. Breed. Crop. Sci..

[30] Yatung T., Dubey R.K., Singh V., Upadhyay G. (2014). Genetic diversity of chilli (*Capsicum annuum* L.) genotypes of India based on morpho-chemical traits. AJCS.

[31] Bonny S. (2011). L’agriculture écologiquement intensive: Nature et défis. Cah. Agric..

[32] Tripodi P., Ficcadenti N., Rotino G.L., Festa G., Bertone A., Pepe A., Caramanico R., Migliori C.A., Spadafora D., Schiavi M. (2019). Genotypic and environmental effects on the agronomic, health-related compounds and antioxidant properties of chilli peppers for diverse market destinations. J. Sci. Food Agric..

[33] Sonneveld C., Voogt W. (2009). Plant Nutrition of Greenhouse Crops.

[34] Yadav R.K., Sangwan R.S., Sabir F., Srivastava A.K., Sangwan N.S. (2014). Effect of prolonged water stress on specialized secondary metabolites, peltate glandular trichomes, and pathway gene expression in *Artemisia annua* L.. Plant. Physiol. Bioch..

[35] Krasteva L., Todorova T. (2003). The Bulgarian Solanaceae collections. Compilers. Solanaceae Genetic Resources in Europe. Report of two Meetings-21 September 2001, Nijmegen, The Netherlands/22 May 2003, Skierniewice, Poland.

