# Supplementary material for: Agro-Morphological Characterization of Sicilian Chili Pepper Accessions for Ornamental Purposes
Source: Plants (Basel). 2020 Oct 21;9(10):1400. doi: 10.3390/plants9101400 (PMC7588915; doi:10.3390/plants9101400)
Supplement: Supplementary file 1 [file plants-09-01400-s001.pdf]

Table S1. Analysis of variance and mean comparisons for plant height (PH), plant canopy width (PCW), PH/PCW ratio and plant visual quality of 34 chili pepper accessions grown into different pot dimensions.

Treatments	PH (cm)	PCW (cm)	PH/PCW ratio	Plant visual quality (1-9)
Pot diameter (cm)				
10	13.1 c	18.5 c	1.04 a	3.1 b
14	24.6 b	28.0 b	0.88 b	6.2 a
18	30.2 a	39.7 a	0.77 c	6.3 a
20	31.2 a	39.7 a	0.79 c	5.7 a
Accession				
A1	27.2 bc	27.2 b	1.02 ab	5.8 ab
A2	18.5 c	24.2 b	0.78 bc	6.0 ab
A3	35.6 ab	40.0 ab	0.92 bc	5.7 ab
A4	30.8 ab	28.3 ab	1.17 ab	3.7 b
A5	38.8 ab	35.5 ab	1.12 ab	3.3 b
A6	15.8 c	23.9 b	0.68 c	4.1 b
A7	29.8 ab	31.2 ab	0.97 b	5.1 ab
A8	18.5 c	28.5 ab	0.67 c	5.6 ab
A9	26.0 bc	26.5 b	1.02 b	5.0 ab
A10	31.4 ab	35.3 ab	0.94 bc	4.3 b
A11	29.5 bc	21.1 ab	0.93 bc	4.9 ab
A12	23.0 bc	26.9 b	0.88 bc	5.7 ab
A13	39.5 a	44.5 ab	0.94 bc	4.0 b
A14	20.7 bc	27.0 b	0.82 bc	4.9 ab
A15	24.2 bc	39.4 ab	0.66 c	5.1 ab
A16	19.6 c	29.1 ab	0.71 c	5.9 ab
A17	28.3 bc	42.9 ab	0.70 c	5.7 ab
A18	29.5 bc	24.0 b	1.27 ab	4.9 ab
A19	28.8 bc	29.5 ab	0.99 b	5.6 ab
A20	17.6 c	26.8 b	0.69 c	4.4 b
A21	18.5 c	31.8 ab	0.65 c	6.7 ab
A22	30.4 ab	40.0 ab	0.79 bc	6.1 ab
A23	29.9 ab	28.2 ab	1.08 ab	5.8 ab
A24	20.0 bc	26.5 b	0.77 bc	5.3 ab
A25	27.1 bc	28.5 ab	0.95 bc	5.0 ab
A26	24.4 bc	28.9 ab	0.86 bc	6.0 ab
A27	25.2 bc	37.7 ab	0.69 c	5.4 ab
A28	30.5 ab	33.1 ab	0.95 bc	4.3 b
A29	21.1 bc	33.5 ab	0.65 c	7.4 a
A30	19.3 c	24.8 b	0.82 bc	3.7 b
A31	30.7 ab	30.3 ab	1.06 ab	5.9 ab
A32	22.9 bc	35.1 ab	0.66 c	6.4 ab
A33	34.5 ab	40.3 ab	0.89 bc	6.7 ab
A34	25.2 b	28.7 ab	0.91 bc	6.4 ab
Significance				
Pot (P)	**	**	**	**
Accession (A)	**	**	**	**
P × A	**	**	**	**

Data within a column followed by the same letter are not significantly different at $p \leq 0.05$ according to Tukey HSD Test. The significance is designated by asterisks as follows: **, statistically significant differences at p-value below 0.01.

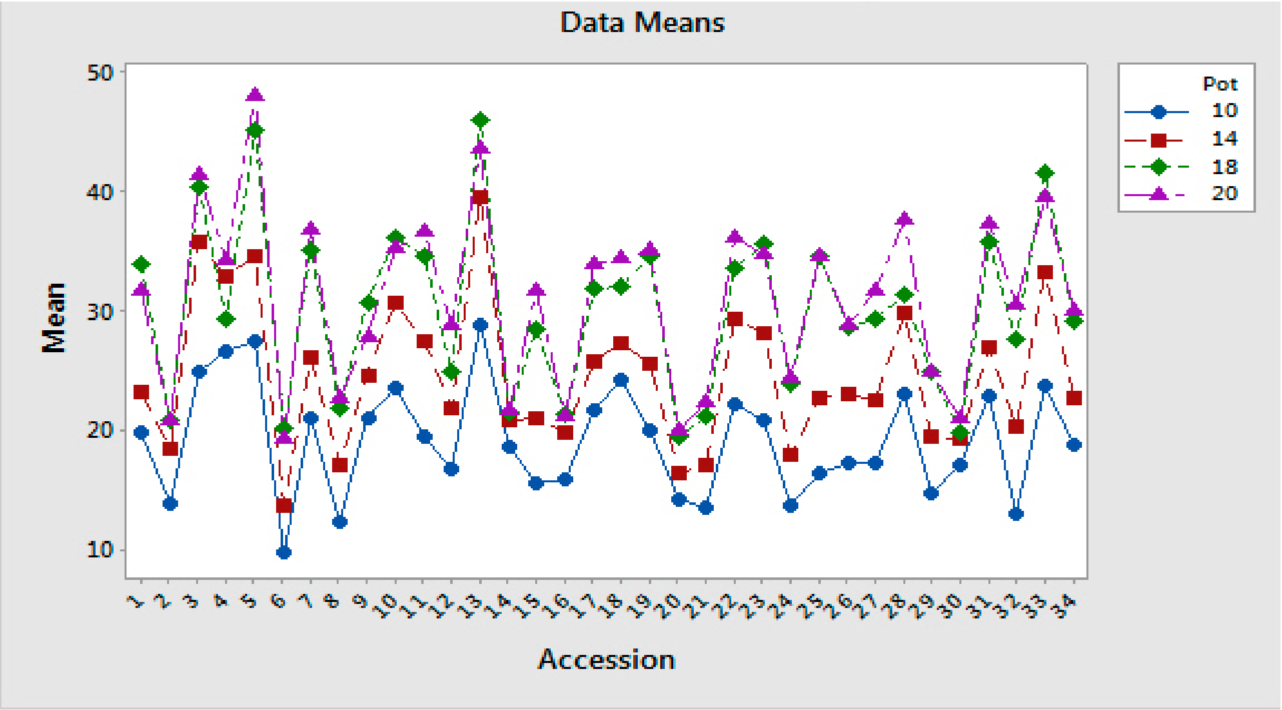

Figure S1. Interaction pot diameter × accession for PH.
1 = accession A1; 2 = accession A2; 3 = accession A3; 4 = accession A4; 5 = accession A5; 6 = accession A6; 7 = accession A7; 8 = accession A8; 9 = accession A9; 10 = accession A10; 11 = accession A11; 12 = accession A12; 13 = accession A13; 14 = accession A14; 15 = accession A15; 16 = accession A16; 17 = accession A17; 18 = accession A18; 19 = accession A19; 20 = accession A20; 21 = accession A21; 22 = accession A22; 23 = accession A23; 24 = accession A24; 25 = accession A25; 26 = accession A26; 27 = accession A27; 28 = accession A28; 29 = accession A29; 30 = accession A30; 31 = accession A31; 32 = accession A32; 33 = accession A33; 34 = accession A34.

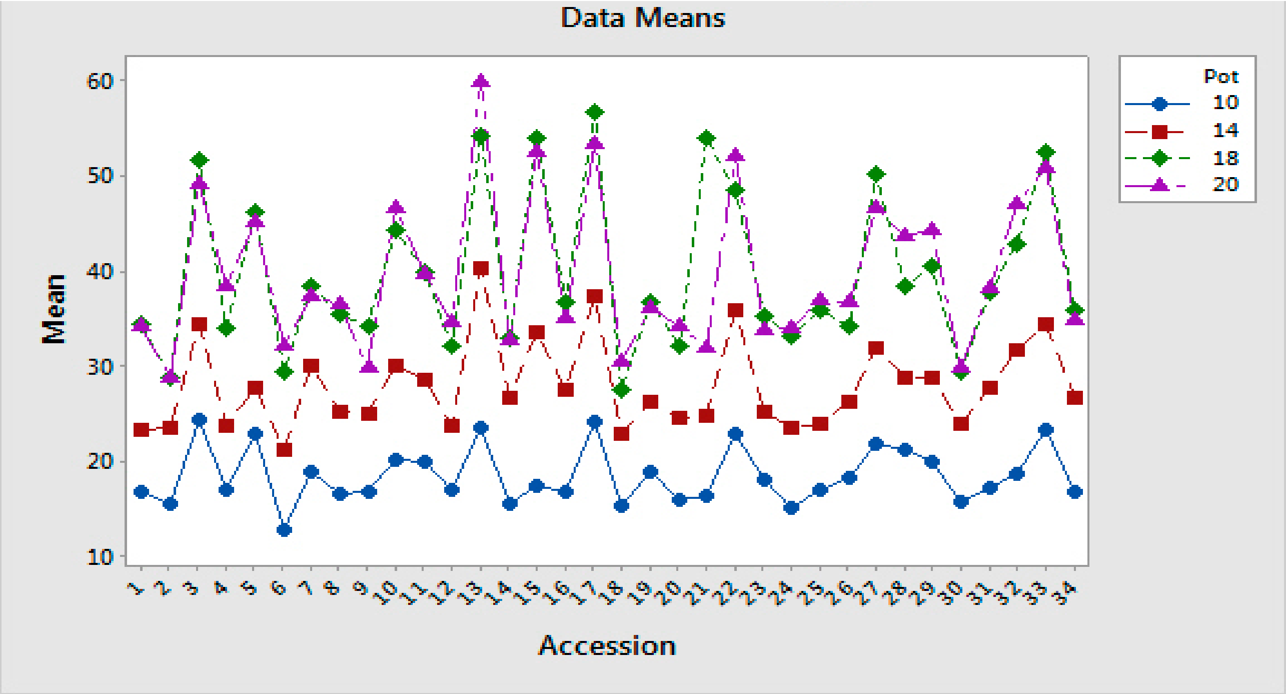

Figure S2. Interaction pot diameter × accession for PCW.
1 = accession A1; 2 = accession A2; 3 = accession A3; 4 = accession A4; 5 = accession A5; 6 = accession A6; 7 = accession A7; 8 = accession A8; 9 = accession A9; 10 = accession A10; 11 = accession A11; 12 = accession A12; 13 = accession A13; 14 = accession A14; 15 = accession A15; 16 = accession A16; 17 = accession A17; 18 = accession A18; 19 = accession A19; 20 = accession A20; 21 = accession A21; 22 = accession A22; 23 = accession A23; 24 = accession A24; 25 = accession A25; 26 = accession A26; 27 = accession A27; 28 = accession A28; 29 = accession A29; 30 = accession A30; 31 = accession A31; 32 = accession A32 ; 33 = accession A33; 34 = accession A34.

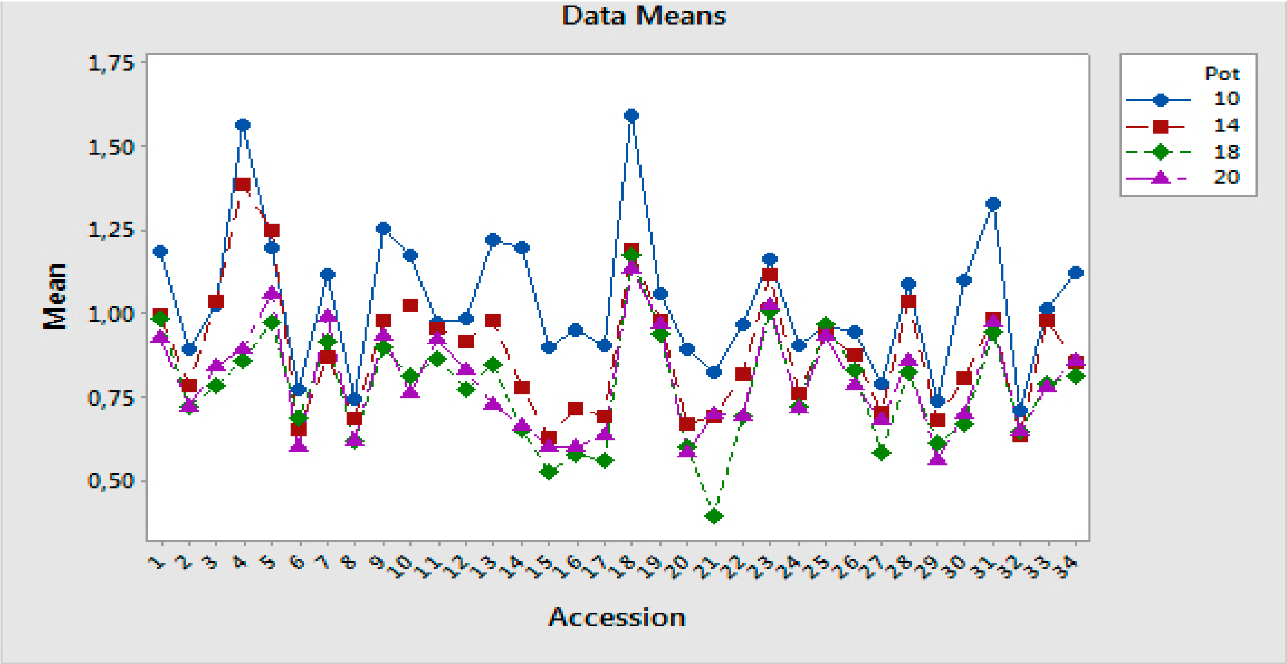

Figure S3. Interaction pot diameter × accession for PH/PCW.
1 = accession A1; 2 = accession A2; 3 = accession A3; 4 = accession A4; 5 = accession A5; 6 = accession A6; 7 = accession A7; 8 = accession A8; 9 = accession A9; 10 = accession A10; 11 = accession A11; 12 = accession A12; 13 = accession A13; 14 = accession A14; 15 = accession A15; 16 = accession A16; 17 = accession A17; 18 = accession A18; 19 = accession A19; 20 = accession A20; 21 = accession A21; 22 = accession A22; 23 = accession A23; 24 = accession A24; 25 = accession A25; 26 = accession A26; 27 = accession A27; 28 = accession A28; 29 = accession A29; 30 = accession A30; 31 = accession A31; 32 = accession A32 ; 33 = accession A33; 34 = accession A34.

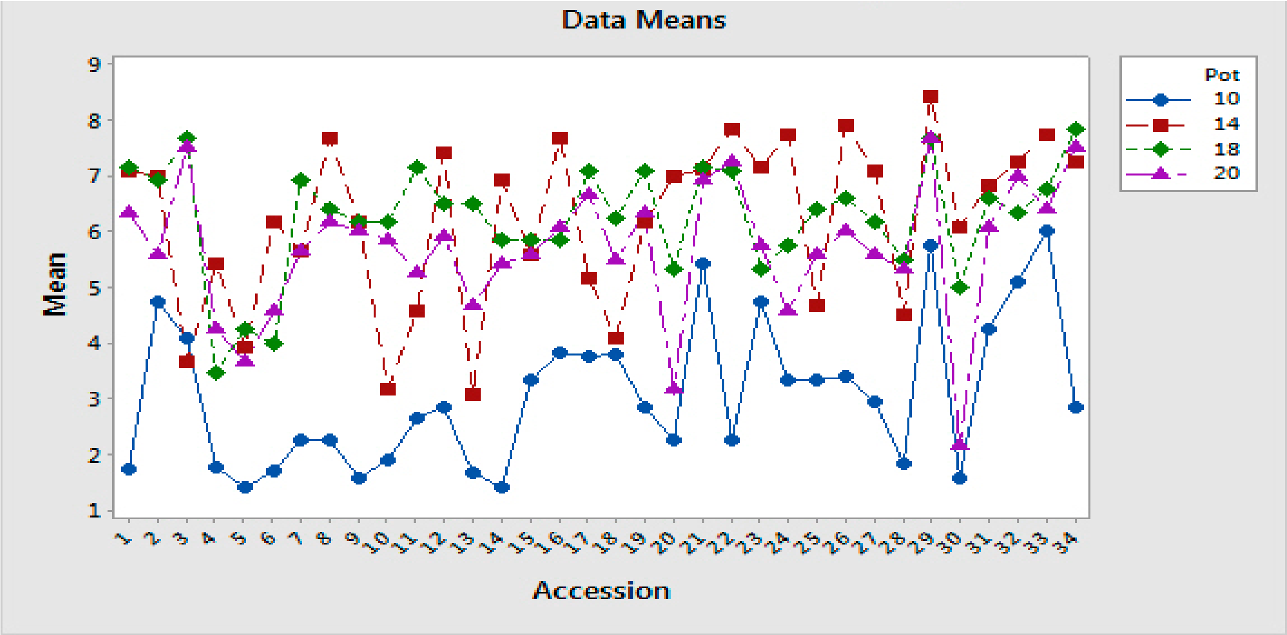

Figure S4. Interaction pot diameter × accession for plant visual quality.
1 = accession A1; 2 = accession A2; 3 = accession A3; 4 = accession A4; 5 = accession A5; 6 = accession A6; 7 = accession A7; 8 = accession A8; 9 = accession A9; 10 = accession A10; 11 = accession A11; 12 = accession A12; 13 = accession A13; 14 = accession A14; 15 = accession A15; 16 = accession A16; 17 = accession A17; 18 = accession A18; 19 = accession A19; 20 = accession A20; 21 = accession A21; 22 = accession A22; 23 = accession A23; 24 = accession A24; 25 = accession A25; 26 = accession A26; 27 = accession A27; 28 = accession A28; 29 = accession A29; 30 = accession A30; 31 = accession A31; 32 = accession A32 ; 33 = accession A33; 34 = accession A34.

Table S2. Significance of two-way ANOVA analysis.

Source of variance	Accession (A)	Year (Y)	A × Y
PH	*	***	***
PCW	***	***	***
PH/PCW	***	***	***
Fruit number	***	***	***
Plant visual quality	***	***	***
First flower emission	***	***	**
Fruiting start	***	***	*
End of harvest	***	***	NS
Fruit length	***	NS	NS
Fruit width	***	NS	NS
Plant growth habit	***	NS	NS
Fruit colour at mature stage	***	NS	NS
Fruit shape	***	NS	NS

The significance is designated by asterisks as follows: ***, statistically significant differences at p-value below 0.001; **, statistically significant differences at p-value below 0.01; *, statistically significant differences at p-value below 0.05; NS, not significant

Table S3. Effect of accessions and year on PH, PCW, PH/PCW, fruit number, plant visual quality, fruit flower emission and fruiting start.

Accessions	PH (cm)		PCW (cm)		PH/PCW		Fruit number		Plant visual quality (1-9)		First flower emission (DAT)		Fruiting start (DAT)	
A1 × 2016	34.00	cd	34.50	gh	0.99	c	132.47	q	7.17	c	35.67	g	42.00	k
A2 × 2016	20.83	hi	28.75	ij	0.72	f	109.75	r	6.92	cd	37.67	e	48.33	e
A6 × 2016	20.17	hi	29.50	ij	0.69	fg	104.03	rs	4.00	h	39.67	d	50.33	cd
A8 × 2016	21.92	gh	35.50	g	0.62	g	78.43	t	6.42	de	40.67	c	48.67	**d**
A12 × 2016	24.83	fg	32.25	hi	0.77	ef	265.60	k	6.50	de	31.67	k	39.00	m
A14 × 2016	21.58	h	32.92	hi	0.65	fg	138.87	q	5.83	f	40.00	d	51.00	c
A15 × 2016	28.42	ef	54.00	b	0.53	h	345.63	i	5.83	f	29.67	m	37.67	n
A16 × 2016	21.33	h	36.83	fg	0.58	gh	325.40	j	5.83	f	35.00	h	40.67	l
A17 × 2016	31.83	d	56.67	a	0.56	gh	113.07	r	7.08	c	40.67	c	53.33	b
A18 × 2016	32.08	d	27.50	j	1.17	b	350.33	i	6.25	e	43.00	a	53.67	ab
A21 × 2016	33.67	cd	48.67	c	0.69	fg	225.37	m	7.08	c	37.67	e	48.33	e
A22 × 2016	23.92	g	33.17	h	0.72	f	428.07	h	5.75	f	42.33	b	50.67	cd
A24 × 2016	34.67	c	35.92	g	0.97	cd	245.93	l	6.42	de	35.00	h	43.67	i
A25 × 2016	29.33	e	50.17	c	0.58	gh	963.63	a	6.17	ef	42.33	b	54.33	a
A27 × 2016	25.00	fg	40.67	ef	0.62	g	621.40	e	7.67	b	43.33	a	53.33	b
A29 × 2016	27.67	ef	42.83	e	0.65	fg	833.97	b	6.33	e	31.33	l	37.67	n
A32 × 2016	41.58	a	52.50	b	0.79	ef	793.70	c	6.75	d	36.67	f	52.67	b
A33 × 2016	29.17	e	35.92	g	0.81	e	224.03	m	7.83	b	36.00	g	47.33	f
G1 × 2016	21.17	hi	54.00	b	0.39	i	273.23	k	7.17	c	39.00	d	48.67	d
A1 × 2017	33.75	cd	34.58	gh	0.98	c	97.69	s	5.50	f	33.00	j	38.67	m
A2 × 2017	22.17	gh	26.83	jk	0.83	de	81.65	t	6.58	de	34.33	i	44.33	i
A6 × 2017	12.97	j	21.81	l	0.59	gh	76.69	tu	5.00	g	36.00	g	47.00	fg
A8 × 2017	22.67	gh	32.58	hi	0.70	f	64.91	u	5.08	g	38.00	e	43.33	j
A12 × 2017	31.42	de	27.83	j	1.13	b	197.54	no	7.17	c	29.00	n	36.00	o
A14 × 2017	27.25	ef	29.50	ij	0.93	cd	102.40	rs	6.00	ef	37.00	e	46.33	g
A15 × 2017	31.83	d	38.83	f	0.82	e	252.56	l	7.58	b	27.00	p	34.67	p
A16 × 2017	23.75	g	30.75	ij	0.77	ef	241.72	l	8.17	ab	32.00	k	36.67	o
A17 × 2017	34.92	c	49.17	c	0.71	f	84.47	t	6.17	ef	38.00	e	49.33	d
A18 × 2017	39.25	b	26.33	jk	1.49	a	262.75	kl	4.58	g	40.00	d	50.67	cd
A21 × 2017	38.33	b	45.25	d	0.85	de	162.36	p	7.50	bc	34.67	h	45.33	h
A22 × 2017	23.83	g	31.33	hi	0.76	ef	317.38	j	7.58	b	39.00	d	47.33	f
A24 × 2017	26.83	f	26.83	jk	1.00	c	187.78	o	6.58	de	32.00	k	41.33	kl
A25 × 2017	26.50	f	38.67	f	0.68	fg	722.73	d	8.42	a	40.00	d	50.67	cd
A27 × 2017	26.58	f	33.67	gh	0.79	ef	474.54	g	7.75	b	39.00	d	48.67	d
A29 × 2017	27.50	ef	39.08	f	0.71	f	618.81	e	8.42	a	27.67	o	34.67	p
A32 × 2017	38.92	b	43.42	de	0.90	d	601.94	f	7.75	b	34.00	i	50.00	d
A33 × 2017	30.33	de	34.75	gh	0.88	de	161.36	p	6.50	de	33.00	j	45.00	h
G1 × 2017	19.17	i	24.58	k	0.78	ef	204.26	n	7.17	c	35.67	g	46.00	g

Data within a column followed by the same letter are not significantly different at $p \leq 0.05$ according to Tukey HSD Test.
